# Crocin Ameliorates Diabetic Nephropathy through Regulating Metabolism, CYP4A11/PPARγ, and TGF-β/Smad Pathways in Mice

**DOI:** 10.2174/0113892002257928231031113337

**Published:** 2023-12-30

**Authors:** Wei Chen, Jinhao Su, Yubin Liu, Tianmei Gao, Xiaohui Ji, Hanzhou Li, Huajun Li, Yuansong Wang, Hui Zhang, Shuquan Lv

**Affiliations:** 1 Department of Endocrinology, Cangzhou Hospital of Integrated Traditional Chinese Medicine and Western Medicine of Hebei Province Affiliated to Hebei University of Chinese Medicine, Cangzhou, China;; 2 Qingxian Branch of Cangzhou Hospital of Integrated Traditional Chinese Medicine and Western Medicine of Hebei Province Affiliated to Hebei University of Chinese Medicine, Cangzhou, China;; 3 Chengde Medical University, Chengde, China

**Keywords:** Diabetic nephropathy, crocin, traditional chinese medicine, arachidonic acid metabolism, CYP4A11/PPARγ pathway, TGF-β/Smad pathway

## Abstract

**Introduction:**

Crocin is one of the main components of *Crocus sativus* L. and can alleviate oxidative stress and inflammation in diabetic nephropathy (DN). However, the specific mechanism by which crocin treats DN still needs to be further elucidated.

**Method:**

In the present study, a mouse model of DN was first established to investigate the therapeutic effect of crocin on DN mice. Subsequently, non-targeted metabolomics techniques were used to analyze the mechanisms of action of crocin in the treatment of DN. The effects of crocin on CYP4A11/PPARγ and TGF-β/Smad pathway were also investigated.

**Result:**

Results showed that crocin exhibited significant therapeutic and anti-inflammatory, and anti-oxidative effects on DN mice. In addition, the non-targeted metabolomics results indicated that crocin treatment affected several metabolites in kidney. These metabolites were mainly associated with biotin metabolism, riboflavin metabolism, and arachidonic acid metabolism. Furthermore, crocin treatment upregulated the decreased levels of CYP4A11 and phosphorylated PPARγ, and reduced the increased levels of TGF-β1 and phosphorylated Smad2/3 in the kidneys of DN mice.

**Conclusion:**

In conclusion, our study validated the considerable therapeutic, anti-inflammatory, and anti-oxidative impacts of crocin on DN mice. The mechanism of crocin treatment may be related to the regulation of biotin riboflavin and arachidonic acid metabolism, the activation of CYP4A11/PPARγ pathway, and the inhibition of TGF-β/Smad pathway in the kidney.

## 1 INTRODUCTION

Diabetes mellitus is a major public health issue throughout the world today. Research has shown that 20-40% of diabetic patients develop diabetic nephropathy (DN) [1], which is a serious complication of diabetes mellitus and the main risk factor of chronic renal failure and end-stage renal disease [2]. The pathogenesis of DN development is complex and multifactorial, with the involvement of many pathways and metabolites [3]. TGF-β contributes to reactive oxygen species (ROS) production in mesangial cells exposed to high glucose levels through NADPH oxidase mediation. The sustained increase and activation of TGF-β, driven by ROS production, lead to excessive remodeling of the extracellular matrix in the mesangium and promote fibrotic processes in the tubular interstitium [3]. Currently, the main preventive and treatment methods for DN in clinical practice mainly include the control of blood glucose, blood pressure and blood lipid levels and supplementation of vitamins and minerals. These measures enable the alleviation of certain clinical symptoms in patients but do not provide ideal therapeutic effects [4-6]. Specialty drugs such as Thiazolidinediones have many attractive benefits, such as regulating blood glucose, enhancing insulin sensitivity, and protecting the kidneys from diabetic damage. However, its serious side effects, including fluid retention,cardiovascular complications, hepatotoxicity, and bone fractures, greatly limit its use in clinical practice [7, 8]. For patients with diabetes and progressive chronic kidney disease, renal transplantation is the best mode of renal replacement therapy and offers long-term survival advantages. However, immune rejection and short-term mortality remain significant drawbacks that cannot be ignored [9]. Therefore, the development of novel drugs for the treatment of DN remains of great importance.

*Crocus sativus* L. is a traditional Chinese herbal medicine often used as a sedative, hypnotic, tonic and expectorant [10]. In clinical practice, *Crocus sativus* L. is widely used in the treatment of conditions such as diabetes, Alzheimer's disease, depression, anxiety, cardiovascular disease, learning and memory disorders, and cancer [11]. A systematic review and meta-analysis of randomized controlled trials found that *Crocus sativus* L. significantly improved kidney function relative to placebo [12]. These studies suggest a potential kidney-protective effect of *Crocus sativus* L. However, there is still a blank on the in-depth mechanism of how *Crocus sativus* L. treats DN. Crocin is one of the key components of *Crocus sativus* L. and provides various pharmacological effects, including hypotensive, hypolipidemic, anti-thrombotic and anti-atherosclerotic effects [13, 14]. Many studies have found that crocin could alleviate the pathophysiological changes associated with DN. A Study has shown that crocin alleviates DN-related oxidative stress and inflammation [15]. *In vitro* study has also shown that crocin protects renal epithelial cells from high glucose-induced injury [16]. Recent studies have revealed that crocin inhibits the epithelial-mesenchymal transition in gastric cancer cells, a pathologic alteration that is equally important in DN [17]. However, there is a lack of in-depth research on the exact mechanisms of crocin on DN. Further exploration is necessary to determine the mechanisms by which crocin delays the progression of DN and whether these mechanisms are associated with certain pathways.

In the present study, a mouse model of DN was first established using a high-sugar, high-fat diet (HFD) combined with streptozotocin (STZ) to investigate the therapeutic, anti-oxidative, and anti-inflammatory effects of crocin on DN mice. Subsequently, non-targeted metabolomics techniques were used to analyze the mechanisms of action of crocin in the treatment of DN. Finally, the changes in pathways related to the altered metabolites were verified to elucidate the deep mechanisms between crocin and DN.

## 2 METHODS

### 2.1 Animals and Materials

Sixty healthy male Specific-pathogen-free (SPF)-grade C57BL/6 mice weighing 20-22 g each were purchased from Beijing HFK Bioscience Co., Ltd. (Animal license number: SCXK (Beijing) 2019-0008). Before model construction, the mice were acclimatized for one week at a room temperature of 21 ± 2°C, relative humidity of 50-60%, and 12 h light/12 h dark cycle. The mice were provided *ad libitum* access to chow and water and housed in groups of five per cage. All animal experiments were approved by the Ethics Committee of Hebei University of Chinese Medicine (Approval no. CZX2021-KY-026) and performed in accordance with the Guide for the Care and Use of Laboratory Animals published by the National Institutes of Health. Information on the reagents and kits used in this study is included in the supplementary material.

### 2.2 Establishment of Mouse Model of DN

After one week of acclimatization, the mice were fed a HFD, consisting of 21% fat, 34% sucrose, 0.15% cholesterol, and 44.85% standard chow for eight weeks. At the end of week 8, the mice fasted for 12 h and subsequently were subjected to intraperitoneal injections of STZ (30 mg/kg, dissolved in 0.1 mol/L sodium citrate buffer, pH=4.5). The control group received intraperitoneal injections of an equivalent volume of vehicle. Blood samples were taken from the tail vein for the measurement of random blood glucose levels 72 hours after injection, with ≥16.7 mmol/L used as the criterion for the successful establishment of the T2DM model. Subsequently, rearing of the mice was continued and the 24h urine total protein (UTP) content was measured weekly. Blood glucose level ≥16.7 mmol/L, positive urine glucose test, and positive UTP test were used as the criteria for successful DN model establishment.

### 2.3 Animal Grouping and Drug Administration Regimens

Upon establishment of the mouse model, the mice were divided using a random number table into five groups of 10 mice each: model group, irbesartan (IRB) group, low-dose crocin (Crocin10) group, medium-dose crocin (Crocin20) group, and high-dose crocin (Crocin40) group. Groups of mice received the following treatments by intragastric administration for 28 consecutive days from 2 p.m. to 4 p.m. each day: (1) Model and control groups: 0.2 mL of saline/d; (2) IRB group: IRB 50 mg/kg/d [18]; (3) Crocin10, Crocin20, and Crocin40 groups: crocin 10 mg/kg/d, 20 mg/kg/d, and 40 mg/kg/d, respectively [19]. Fasting blood glucose (FBG) and body weight of the mice were measured every week.

### 2.4 Biochemical Marker Measurement

24 h urine samples were collected from the mice using metabolic cages after four weeks of intervention with crocin. The samples were centrifuged at 4000 g for 10 min, and 24 h UTP was measured according to the instructions provided with the test kit. Blood samples were collected from the heart of each mouse and centrifuged at 500 g for 15 min. The resultant serum was collected and subjected to measurement of creatinine (Cr) and blood urea nitrogen (BUN) levels in accordance with the instructions provided with the assay kits.

### 2.5 Histopathological Staining of Renal Tissue

After urine and blood sample collection, mice were sacrificed. The left kidney was obtained from the mice, fixed in 4% paraformaldehyde solution, dehydrated, embedded in paraffin, and sectioned. The sections were separately subjected to hematoxylin and eosin (HE) staining and periodic acid-Schiff (PAS) staining, and histopathological changes in the tissue samples were observed under an optical microscope. Besides, the positive expressions of phospho-suppressor of mothers against decapentaplegic (Smad) 2, and phospho-Smad 3 in kidney were detected using immunofluorescence, as described previously [15]. The co-localization of phospho-Smad 2, phospho-Smad 3 and nucleus was quantified by Image J. Manders' Coefficients were calculated for the quantification of co-localization.

### 2.6 Measurement of Antioxidant-Related Markers in Renal Tissue

After the left kidney collection, the right kidney was rapidly removed. Renal tissue was obtained from the kidneys, washed three times with cold PBS, and cut into small pieces. 0.1 g of tissue was then added to 900 μL of saline and subjected to low-temperature homogenization. After centrifugation at 1000 g for 15 min, the supernatant was collected. Oxidative stress markers, including superoxide dismutase (SOD) activity, glutathione peroxidase (GSH-Px) activity, and malondialdehyde (MDA) level, were measured in accordance with instructions provided with the assay kit.

### 2.7 Measurement of Inflammatory Factors in Renal Tissue Homogenates

Interleukin (IL) -6, IL-1β, and tumor necrosis factor α (TNF-α) levels in renal tissue homogenates of the various groups were measured using the enzyme-linked immunosorbent assay (ELISA). Testing was separately performed in accordance with the instructions of the various assay kits. Each sample was tested in triplicate wells, and the standards provided with the assay kits were separately added for the preparation of standard curves. After incubation, the wells were washed, incubated for another 30 min with the corresponding enzyme-conjugated secondary antibody, and washed again. This was followed by the addition of a chromogenic medium and the measurement of absorbance at 405 nm using a plate reader for the calculation of IL-6, IL-1β, and TNF-α concentrations.

### 2.8 Metabolomics Analysis

100 mg of renal tissue ground in liquid nitrogen was mixed with 500 μL of 80% methanol. The mixture was subjected to vortex shaking and cooled in an ice bath for 5 min. Subsequently, the mixture was centrifuged at 15000 g at 4°C for 20 min, and the supernatant was collected, diluted with mass spectrometry-grade water to 53% methanol, and centrifuged again at 15000 g at 4°C for 20 min. The supernatant obtained from the second centrifugation was collected. 20 μL of each sample was drawn and mixed for use as a quality control (QC) sample. Details regarding the LC-MS conditions and the data acquisition, processing and analysis steps have been provided in the supplementary material.

### 2.9 Western Blotting

Total protein was extracted from renal tissue, and protein concentration was measured using the bicinchoninic acid (BCA) assay. Subsequently, a protein loading buffer was added, and the mixture was heated at 99°C for 5 min to allow sufficient protein denaturation. The protein samples of the various groups were subjected to protein separation by sodium dodecyl-sulfate polyacrylamide gel electrophoresis. After separation, the proteins were transferred to a polyvinylidene difluoride membrane, blocked for 2 h with 5% skim milk, and separately incubated overnight at 4°C with the primary antibodies of cytochrome P450 (CYP) 4A11, peroxisome proliferator-activated receptor gamma (PPARγ), phospho-PPARγ, transforming growth factor beta (TGFβ) 1, suppressor of mothers against decapentaplegic (Smad) 3, phospho-Smad3, Smad2, phospho-Smad2, and β-actin. The membrane was then washed thrice and the secondary antibody was added for incubation at room temperature for 2 h. After incubation, the membrane was washed thrice, sufficiently reacted with an enhanced chemiluminescence reagent, and exposed using an automatic gel imaging system to obtain the protein bands. The grayscale values of the protein bands were analyzed using ImageJ software for the calculation of relative protein expression levels.

### 2.10 Statistical Analysis

Statistical analysis was performed using SPSS. Comparisons between two groups were performed using the *t* test. Differences were considered statistically significant when *p<*0.05.

## 3 RESULTS

### 3.1 Effects of Crocin on Body Weight, Blood Glucose, and Renal Function Markers of Mice

**Fig. (1):**
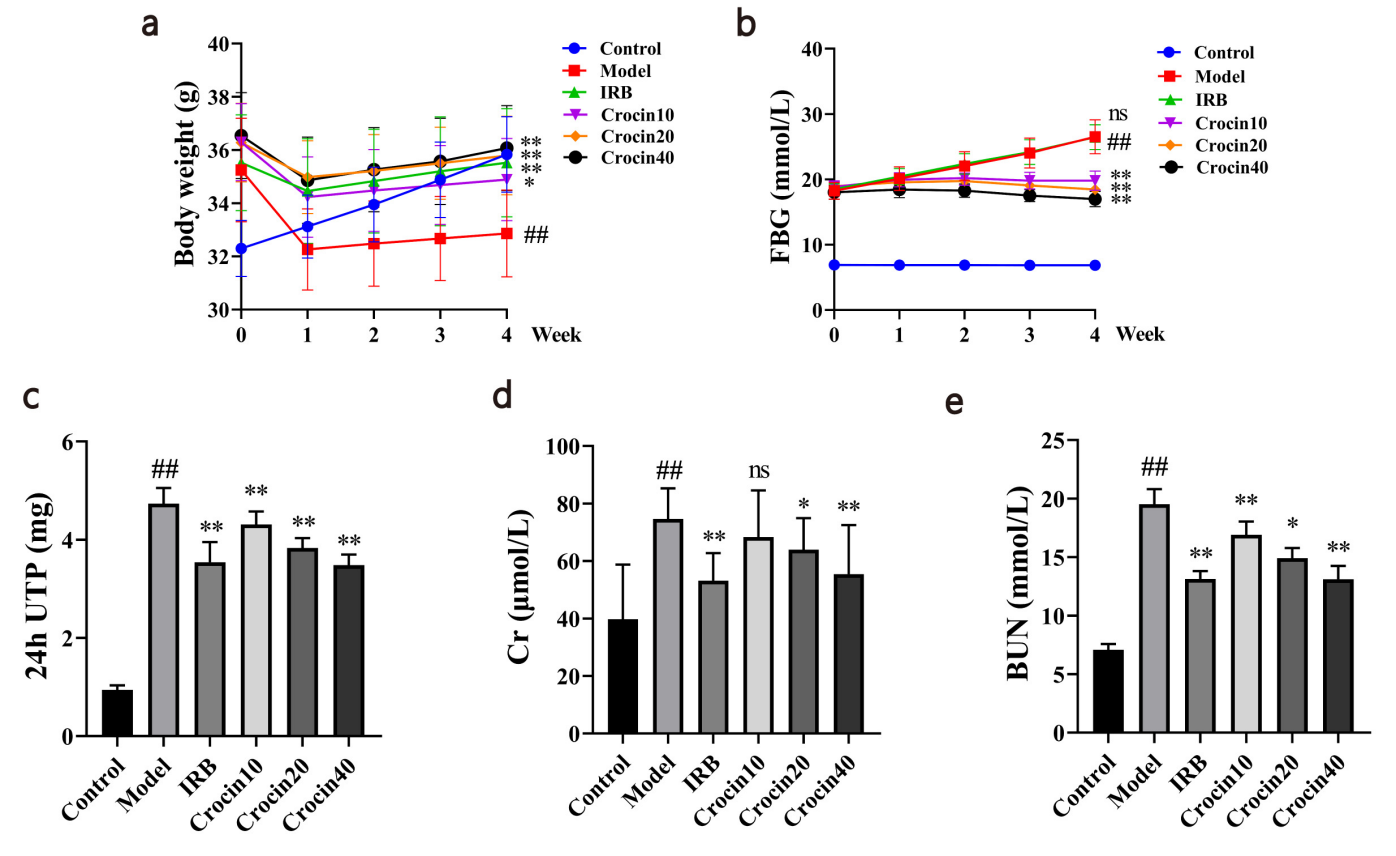
Crocin had a therapeutic effect on STZ-induced DN mice. The study involved administering various doses of crocin and IRB to mice starting from the first day of establishing the DN model. On the 42^nd^ day, all mice were euthanized, and their responses were analyzed. The effects of crocin on body weight (**a**) and FBG (**b**) change curves of each group were examined. The changes in kidney function 24h UTP (**c**), Cr (**d**) and BUN (**e**) after crocin treatment were also evaluated. Control, Model, IRB, Croncin10, Croncin20 and Croncin40 (n = 10 per group) groups. Data are presented as the mean ± standard deviation. ##: *p<*0.01 as compared to the Control group; *: *p<*0.05 as compared to the Model group; **: *p<*0.01 as compared to the Model group.

Changes in body weight and FBG were observed every week after drug administration. Upon completion of drug administration, the model group showed a significant decrease in body weight compared with the control group, while the IRB, Crocin10, Crocin20, and Crocin40 groups exhibited a significant increase in body weight compared with the model group (Fig. **1a**). The model group showed a significant rise in FBG compared with the control group, and the IRB, Crocin10, Crocin20, and Crocin40 groups exhibited a significant decrease in FBG compared with the model group (Fig. **1b**).

Results of renal function measurement revealed that the serum Cr, BUN and 24 h UTP levels of the model group were significantly increased compared with the control group. It was found that intervention with IRB and medium and high doses of crocin significantly decreased the 24 h UTP, serum Cr, and BUN levels of DN mice compared with the model group (Fig. **1c**-**e**). Intervention with IRB and high-dose crocin significantly reduced the 24 h UTP and serum BUN levels, while low-dose crocin intervention was also capable of significantly reducing BUN level (Fig. **1c**-**e**).

**Fig. (2):**
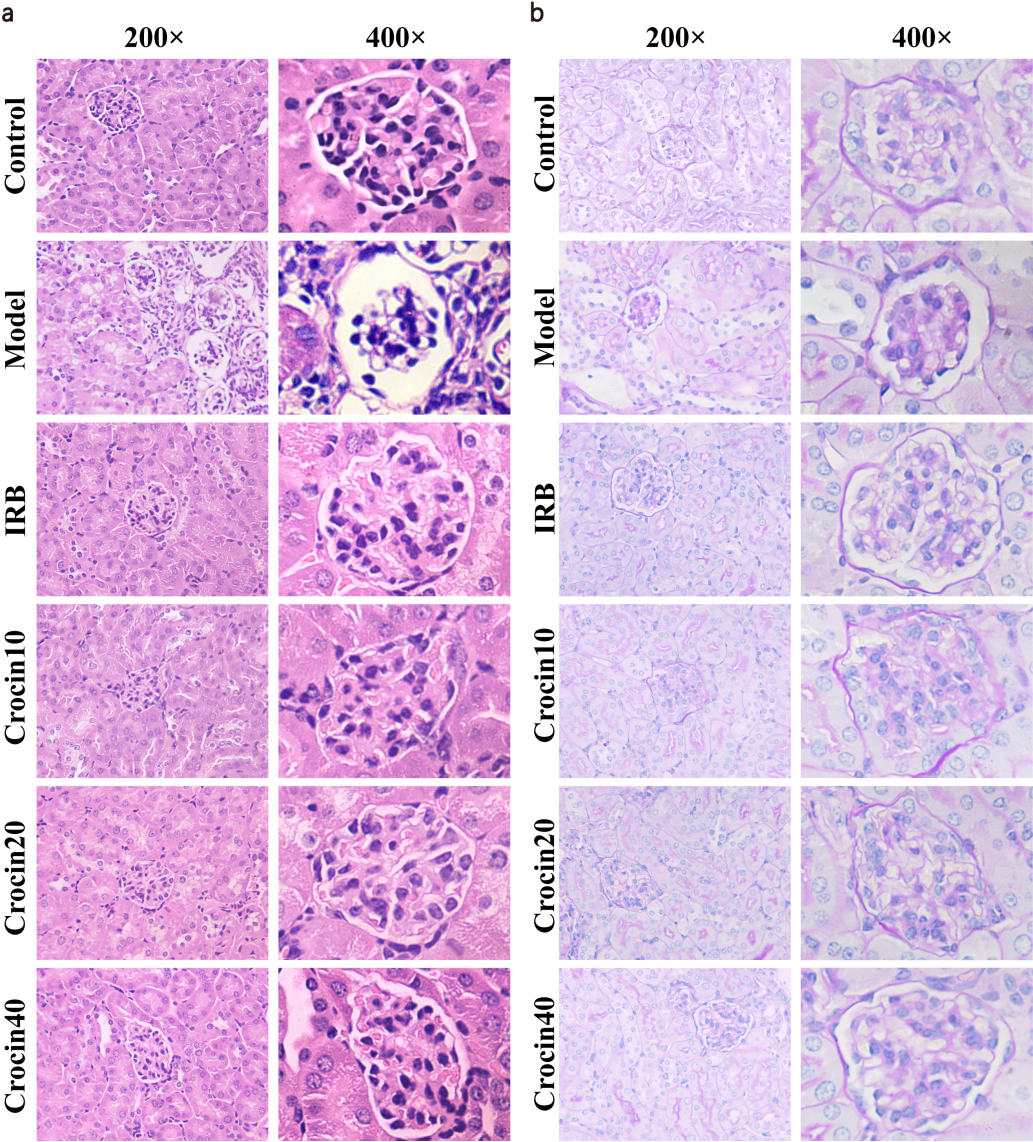
Crocin ameliorated the pathological changes in kidney of DN mice. HE staining (**a**) and PAS staining (**b**) of kidney showed crocin reduced the pathological changes in DN mice.

HE staining and PAS staining results showed that the morphology of glomeruli in the model group mice was irregular, with uneven width of the glomerular capsule, and the mesangial matrix was abundant and unevenly distributed (Fig. **2a**-**b**). HE staining also revealed inflammatory cell infiltration in the renal interstitium and glomeruli of the model mice. PAS staining also indicated that a large amount of collagen formation occurred in the renal cysts of the model mice. After treatment with IRB or crocin, the above symptoms were improved, with the most significant improvement observed in the IRB and high-dose crocin groups.

### 3.2 Effects of Crocin on Oxidative Stress and Inflammatory Factor Levels

**Fig. (3):**
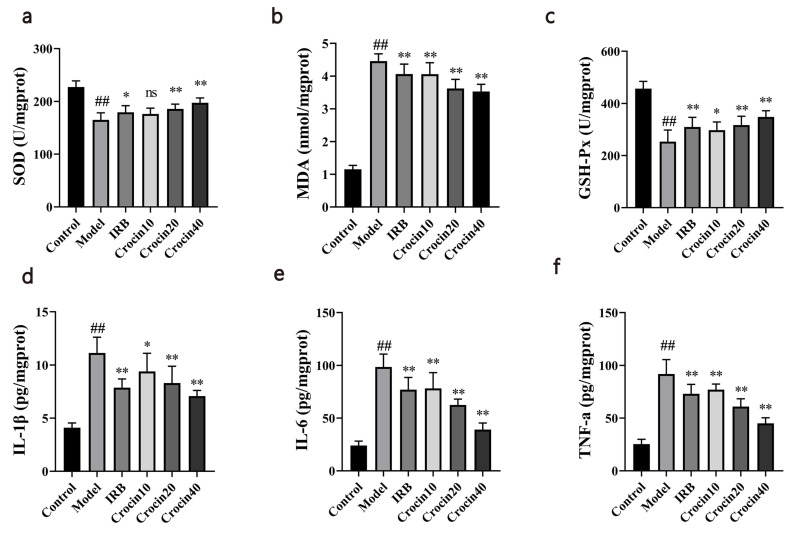
Crocin had anti-oxidative and anti-inflammatory effects on STZ-induced DN mice. To evaluate the anti-inflammatory and anti-oxidative properties of crocin, its effects on DN mice were studied by analyzing oxidative-related factors and pro-inflammatory cytokines in the kidney. (**a-c**) Crocin treatment improved the activities of SOD (**a**) and GSH-Px (**c**), while also reducing the level of MDA (**b**). (**d-f**) Crocin administration to DN mice resulted in a reduction in the levels of IL-1β (**d**), IL-6 (**e**), and TNF-α (**f**) in the kidney. Control, Model, IRB, Croncin10, Croncin20 and Croncin40 (n = 10 per group) groups. Data are presented as the mean ± standard deviation. ##: *p<*0.01 as compared to the Control group; *: *p<*0.05 as compared to the Model group; **: *p<*0.01 as compared to the Model group.

Compared with the control group, the model group exhibited a significant decrease in SOD and GSH-Px activities and a significant increase in MDA concentration. This suggests that DN caused oxidative stress injury in mice. SOD activity was significantly increased in the IRB, Crocin20, and Crocin40 groups, GSH-Px activity was significantly higher in the IRB, Crocin10, Crocin20, and Crocin40 groups, and MDA concentration was significantly lower in the IRB, Crocin10, Crocin20, and Crocin40 groups compared with the model group (Fig. **3a**-**c**). These results suggest that crocin treatment significantly attenuated oxidative stress injury in DN mice.

Compared with the control group, the model group showed significant increases in IL-6, IL-1β, and TNF-α levels. IL-6 level was significantly decreased in the IRB, Crocin10, Crocin20, and Crocin40 groups, IL-1β level was significantly lower in the IRB, Crocin10, Crocin20, and Crocin40 groups, and TNF-α level was significantly decreased in the IRB, Crocin10, Crocin20, and Crocin40 groups compared with the model group (Fig. **3d**-**f**). These results suggest that crocin treatment significantly lowered the degree of inflammation in DN mice.

### 3.3 Effects of Crocin on Renal Tissue Metabolites in DN Mice

The results of the analysis of the effects of crocin on oxidative stress and inflammatory levels described above indicate that high-dose crocin caused the greatest improvement in DN mice. Therefore, the control, model and Crocin40 groups were selected for subsequent metabolomics analysis.

**Fig. (4):**
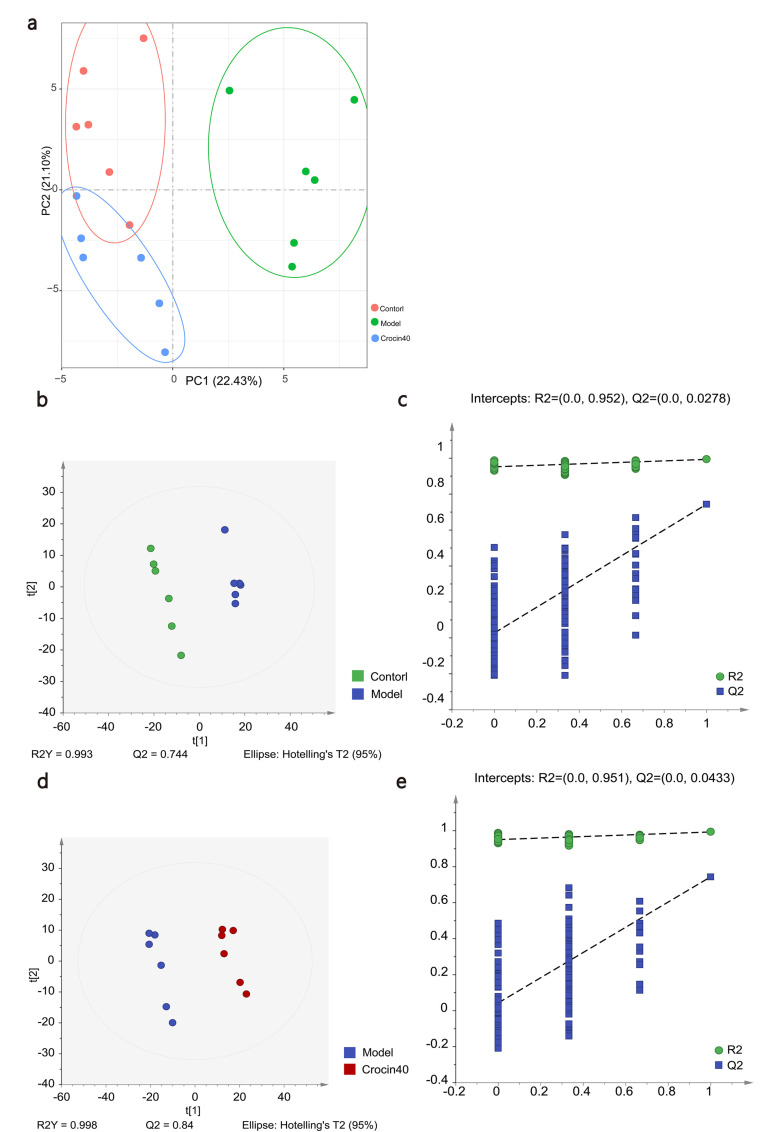
Crocin altered the metabolites of kidney in STZ-induced DN mice. The effects of crocin40 on DN were assessed using multivariate statistical analysis and pathway analysis. (**a**) Score plots of PCA were generated to compare control, model, and crocin40 groups. (**b-e**) Score plots and permutation tests of PLS-DA were performed to compare control and model groups (**b, c**) and model and crocin40 groups (**d, e**). Control, Model, and Crocin40 groups, n=6 per group.

Principal component analysis (PCA) results of samples of the various groups (Fig. **4a**) revealed a clear separation of intergroup samples, which indicates successful model construction in the model group and that crocin exerted certain effects on DN mice. To further elucidate the differences among groups, a supervised partial least-squares discriminant analysis (PLS-DA) model was built for various groups. The PLS-DA model was subsequently validated, with R^2^X and R^2^Y denoting the explanatory ability of the model towards the X- and Y-matrices, respectively, and Q^2^ denoting the predictive power of the model. Results indicated that R^2^Y=0.993, and Q^2^=0.744 when the control group was compared with the model group, and R^2^Y=0.998, and Q^2^=0.84 when the model group was compared with the Crocin40 group. R^2^Y and Q^2^ values >0.5, which demonstrated that the model had good explanatory ability and predictive power (Fig. **4b**-**e**). Therefore, the model is effective and reliable, with good separation achieved among samples of the various groups.

**Fig. (5):**
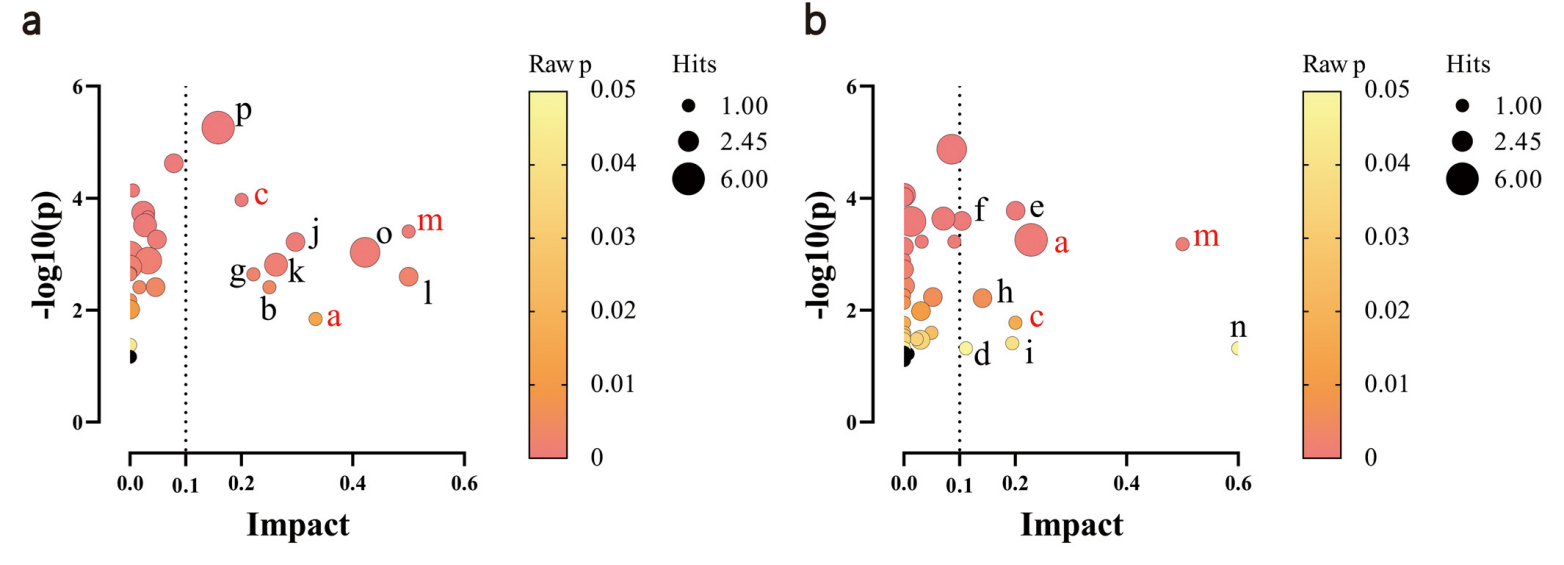
Crocin altered the metabolic pathways of kidney in STZ-induced DN mice. Pathway analysis results were obtained to compare control and model groups (**a**) and model and crocin40 groups (**b**). Common pathways are marked in red. Black bubbles indicate pathways with *p*≥0.05. Name of pathways a: Arachidonic acid metabolism, b: Ascorbate and aldarate metabolism, c: Biotin metabolism, d: Butanoate metabolism, e: Cysteine and methionine metabolism, f: Glycerophospholipid metabolism, g: Histidine metabolism, h: Lysine degradation, i: Nicotinate and nicotinamide metabolism, j: Pentose and glucuronate interconversions, k: Phenylalanine metabolism, l: Phenylalanine, tyrosine and tryptophan biosynthesis, m: Riboflavin metabolism, n: Synthesis and degradation of ketone bodies, o: Tryptophan metabolism, p: Tyrosine metabolism.

The screening criteria for differential metabolites were variable importance in projection (VIP) > 1, *p<*0.05, and fold change (FC) ≥1.2 or ≤ 0.8A total of 130 differential metabolites were screened from the control, model, and Crocin40 groups, as shown in Table **1**. Screened differential metabolites were uploaded to MetaboAnalyst 5.0 (www.metaboanalyst.ca) for metabolic pathway analysis. The 130 differential metabolites of the control, model, and Crocin40 group were enriched in three metabolism-related pathways, namely arachidonic acid metabolism, biotin metabolism, and riboflavin metabolism (Fig. **5a**, **b**).

### 3.4 Effects of Crocin on CYP4A11 and TGFβ-related Protein Expression in DN Mice

**Fig. (6):**
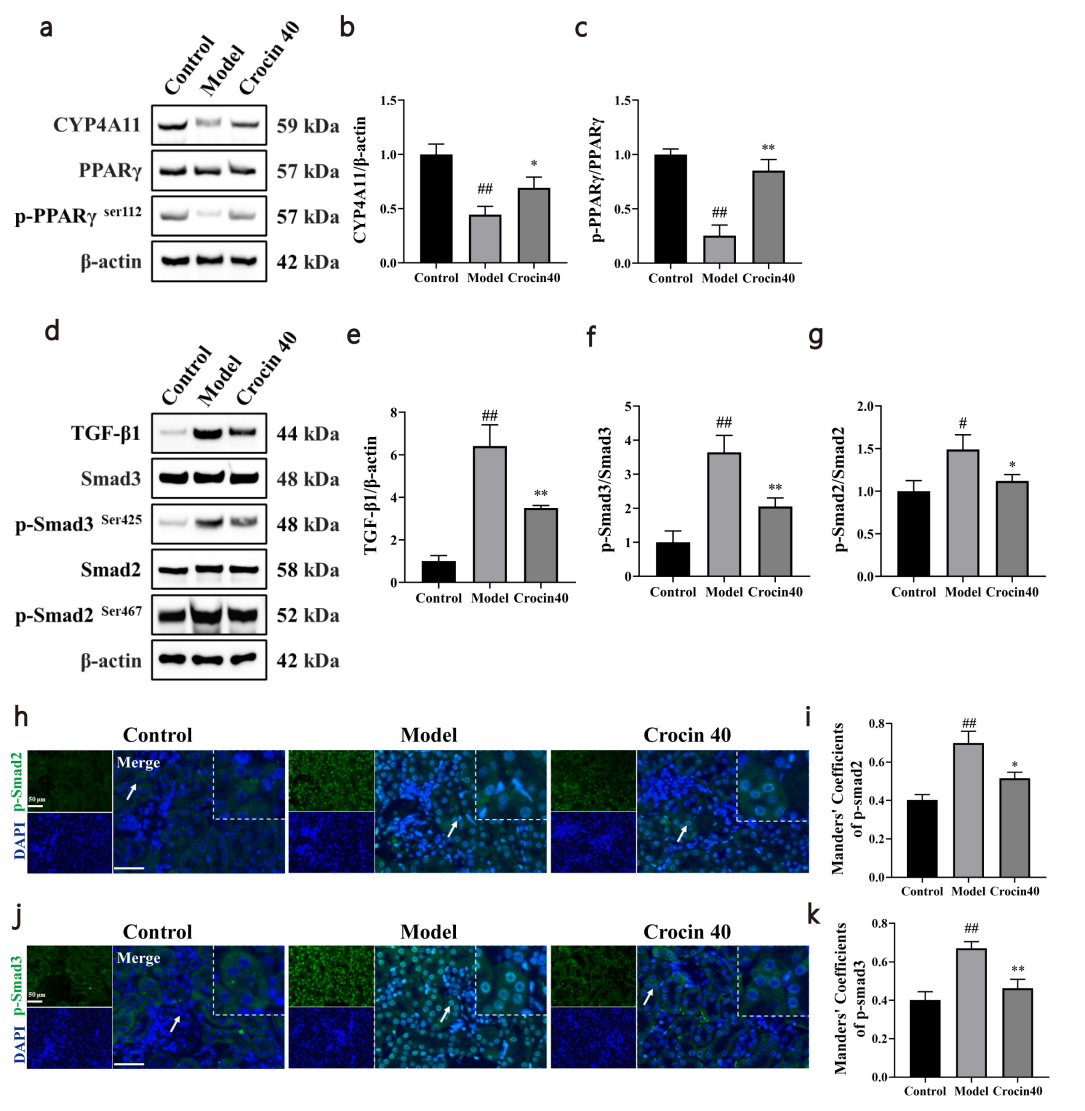
Crocin altered the CYP4A11/PPARγ, TGF-β/Smad signaling pathways in kidney of DN mice. The expression levels of various proteins were examined by western blot analysis and the results were presented as representative blots and histograms. (**a-c**) Crocin elevated CYP4A11 expression levels and increased the phosphorylation level of PPARγ in kidney of DN mice. (**d**-**g**) The administration of crocin resulted in a decrease in the levels of TGF-β1 and the phosphorylation levels of Smad2/3 in the kidney of DN mice. **(h-k)** Immunofluorescence staining showed that crocin treatment reduced the nucleus transition of p-Smad 2 and p-Smad 3 in kidney (400 ×). White arrows indicated the amplified areas. Control, Model, and Crocin40 groups, n=3 per group. Data are presented as the mean ± standard deviation. ##: *p<*0.01 as compared to the Control group; *: *p<*0.05 as compared to the Model group; **: *p<*0.01 as compared to the Model group.

Results of Western blotting indicated that CYP4A11 and phospho-PPARγ protein expression was significantly decreased and TGF-β1, phospho-Smad2 and phospho-Smad3 protein expression were significantly increased in the model group compared with the control group. Results of Western blotting indicated that CYP4A11 and phospho-PPARγ protein expression was significantly increased and TGF-β1, phospho-Smad2 and phospho-Smad3 protein expression were significantly decreased in the Crocin40 group compared with the model group (Fig. **6a**-**g** ). Besides, immunofluorescence showed the nuclear transition of phospho-Smad 2 and phospho-Smad 3. The results showed that the co-localization of phospho-Smad 2, phospho-Smad 3 and nucleus was higher in the kidney in DN mice compared with the mice control group, whereas crocin treatment decreased the co-localization of phospho-Smad 2, phospho-Smad 3 and nucleus in DN mice (Fig. **6h**-**k**).

## 4 DISCUSSION

Animal models established through a combination of HFD feeding and STZ injection have been widely used in research on DN [20]. A prolonged hyperglycemic state elicits compensatory effects in the kidneys, which cause functional and organic damage and ultimately result in the establishment of DN in T2DM model mice [21]. Due to prolonged hyperglycemia, DN may cause muscle damage and significant body weight loss. In the early stages, DN is mainly characterized by a decrease in glomerular filtration rate and an increase in UTP content [22]. With the progression of DN, levels of the renal injury markers Cr and BUN become abnormally increased, and the kidneys exhibit pathological changes such as hypertrophy of glomerular and tubular epithelial cells and thickening of the glomerular and tubular basement membranes. In the present study, the DN mouse model established by a combination of HFD feeding and STZ injection exhibited a significant decrease in body weight and significant increases in FBG, 24 h UTP, Cr, and BUN compared with the control group. Pathological changes were also observed in the kidneys of the DN mice, such as thickening of the glomerular basement membrane, dilation of the renal tubular lumen, inflammatory infiltration, and obvious fibrosis. These results indicate the successful establishment of the DN mouse model. Following intervention with crocin, the body weight, FBG, 24 h UTP, Cr, and BUN of DN mice showed significant recovery, renal glomerular and tubular structures became increasingly normal, and the degree of inflammatory infiltration and fibrosis was decreased. It is therefore, apparent that crocin had a clear therapeutic effect on DN mice. IRB is an angiotensin II receptor antagonist commonly used for the treatment of DN in clinical practice and as a positive control drug in experimental studies [23].

It was observed that crocin increased the levels of the key antioxidant defense enzymes SOD and GSH-Px and decreased the level of MDA. These results demonstrate that crocin possesses a good antioxidant ability. In addition, the levels of pro-inflammatory cytokines in kidney of DN mice decreased after crocin intervention. Both oxidative stress and the inflammatory response serve important roles in DN. Oxidative stress can cause inflammation and the resultant inflammatory response, in turn, exacerbates oxidative stress, thereby inducing changes in renal structure and function [24]. Common inflammatory factors such as IL-6, IL-1β, and TNF-α usually show a trend of increase in DN. The accumulation of many inflammatory factors in the kidneys not only activates the NF-κB pathway and aggravates the inflammatory response but also causes an increase in reactive oxygen species (ROS) and further aggravates oxidative stress [25-27].

Further untargeted metabolomics analysis showed that biotin metabolism, riboflavin metabolism, and arachidonic acid (AA) metabolism were the common metabolic pathways, suggesting that these pathways may be potential routes for the treatment of DN. In biotin metabolism pathway, we found that crocin enabled an increase in decreased biotin levels of the renal tissue of DN mice. Biotin is effective for both types of diabetes, although the mechanisms of hyperglycemia differ [28-30]. Clinical studies have found that high doses of biotin can control blood glucose levels in patients with T1DM or T2DM and prevent diabetic complications [31]. Supplementation of biotin and chromium picolinate to T2DM rats has shown a good anti-diabetic effect [32, 33]. However, there is still insufficient research on the relationship between biotin and diabetes and also a lack of studies of value on biotin and DN. Besides, we found that crocin enabled an increase in decreased vitamin B2 levels of DN mice. Vitamin B2, also known as riboflavin, is a core component of riboflavin metabolism. Research has shown that riboflavin treatment led to a decrease in renal and hepatic function indicators in diabetic mice, which suggests that damage to the renal tubules and glomeruli caused by T2DM had been alleviated [34, 35]. However, studies on the relationship between vitamin B2 and diabetes are extremely scarce and further research is required to elucidate the underlying mechanisms. Furthermore, our results indicated that the decreased levels of AA and 11(12)-EET in the kidneys of DN mice were increased after crocin treatment. AA serves as a precursor for a wide range of metabolites such as prostaglandins, thromboxanes, lipoxins, leukotrienes, hydroxyeicosatetraenoic acids (HETEs), and epoxyeicosatetraenoic acids (EETs) through three metabolic pathways separately regulated by cyclooxygenases (COX), lipoxygenases (LOX), and CYP [36]. Previous research has shown that EETs can alleviate the progression of renal damage associated with hypertension and T2DM [37]. EETs have also been proven to serve a crucial role in cellular injury in the kidneys [38]. The inhibition of EETs induces the production of ROS and mediates high glucose-induced damage to proximal tubular cells by increasing fibrin and collagen IV protein expression and inducing cell hypertrophy [39]. It has been found that blockade of proliferator-activated receptor γ (PPARγ) with GW9662 obliterates EET-mediated anti-inflammatory effects, which suggests that PPARγ is an effector of EETs [40].

Based on the close relationship between AA metabolism, CYP and PPARγ pathway, we further tested the effects of crocin on CYP and PPARγ pathway. We found that crocin treatment upregulated the diminished expression of CYP4A11 and enhanced PPARγ phosphorylation. The CYP4A family is highly expressed in the kidneys and catalyzes the synthesis of 20-HETE and 11(12)-EET [41, 42]. Research has found that CYP4A expression was significantly downregulated in the kidneys of DN rats [43]. Interestingly, previous research has also reported that EETs can inhibit the expression of TGF-β1 target genes and TGF-β1-induced Smad2/3 phosphorylation by promoting PPARγ activation [44-46]. We, therefore, tested the effects of crocin on TGF-β/Smad pathway. Treatment with crocin also reduced the elevated TGF-β1 level and the degree of Smad2/3 phosphorylation in the kidneys of DN mice. Besides, crocin inhibited the nucleus transition of Smad2/3 in the kidney. Many studies have reported that the regulation of PPARγ or the TGF-β/Smad signaling pathway enables the alleviation of DN [47, 48]. Activated TGF-β1 recruits and activates TGFBR1 and subsequently phosphorylates Smad2/3, which enables its translocation into the nucleus for regulation of target gene transcription [49]. This ultimately leads to glomerular hypertrophy and increased production of collagen and fibrin by the glomeruli [50].

## CONCLUSION

To summarize, our study validated the considerable therapeutic, anti-inflammatory, and anti-oxidative impacts of crocin on DN mice. The mechanism of crocin treatment may be related to the regulation of biotin riboflavin and AA metabolism, the activation of CYP4A11/PPARγ pathway, and the inhibition of TGF-β/Smad pathway in the kidney.

## Figures and Tables

**Table 1:** Differential metabolites in kidney after crocin treatment.

**No.**	**Formula**	**RT**	**m/z**	**Metabolites**	**VIP**		**FC**		**Trend**		**Pathway**
**-**	**-**	**-**	**-**	**-**	**M ** ***vs*. C**	**CR *vs*. M**	**M ** ***vs*. C**	**CR *vs*. M**	**M ** ***vs*. C**	**CR *vs*. M**	**-**
1	C_20_ H_32_ O_3_	13.25	319.23	(+/-)11(12)-EET	1.23	1.71	0.21	2.36	↓##	↑*	a
2	C_22_ H_34_ O_4_	12.87	361.24	(+/-)19(20)-DiHDPA	1.48	1.45	1.95	0.50	↑#	↓*	-
3	C_18_ H_34_ O_4_	12.79	313.24	(±)12(13)-DiHOME	1.52	1.40	1.74	0.70	↑##	↓*	-
4	C_9_ H_12_ O_7_ S	7.50	263.02	(3-Methoxy-4-hydroxyphenyl)ethylene glycol sulfate	1.32	1.79	0.28	7.91	↓#	↑**	-
5	C_13_ H_10_ N_2_ O_2_	5.99	225.06	1,3-dipyridin-3-ylpropane-1,3-dione	1.42	1.06	0.71	1.90	↓##	↑**	-
6	C_20_ H_34_ O_5_	11.29	353.23	13,14-dihydro-15-keto prostaglandin D1	1.45	1.61	1.83	0.42	↑#	↓*	-
7	C_20_ H_32_ O_5_	12.26	353.23	13,14-dihydro-15-keto-PGD2	1.83	1.00	1.83	0.41	↑##	↓**	-
8	C_22_ H_40_ O_2_	14.95	335.30	13Z,16Z-docosadienoic acid	1.58	1.57	1.38	0.57	↑#	↓**	-
9	C_20_ H_32_ O_4_	12.78	335.22	15(S)-HpETE	0.89	1.41	1.56	0.35	↑	↓*	a
10	C_20_ H_32_ O_3_	13.91	343.22	16(R)-HETE	1.11	1.74	1.50	0.28	↑	↓**	a
11	C_16_ H_32_ O_3_	14.05	253.22	16-Hydroxyhexadecanoic acid	1.93	1.78	0.37	1.76	↓#	↑*	-
12	C_30_ H_46_ O_4_	13.07	471.35	18-β-Glycyrrhetinic acid	1.77	1.58	0.41	3.20	↓#	↑**	-
13	C_20_ H_30_ O_5_	11.93	331.19	19(R)-hydroxy Prostaglandin A2	1.50	1.30	1.89	0.57	↑#	↓*	-
14	C_10_ H_8_ O	8.86	145.06	1-Naphthol	1.57	1.59	3.11	0.45	↑#	↓*	-
15	C_13_ H_14_ N_2_ O_4_	8.11	263.10	2-(acetylamino)-3-[4-(acetylamino)phenyl]acrylic acid	1.32	1.46	0.64	2.89	↓#	↑**	-
16	C_17_ H_34_ O_4_	13.61	325.24	2,3-dihydroxypropyl 12-methyltridecanoate	1.80	1.57	2.39	0.47	↑#	↓*	-
17	C_19_ H_36_ O_4_	14.21	329.27	2,4-dihydroxyheptadec-16-en-1-yl acetate	1.95	1.62	1.72	0.40	↑#	↓**	-
18	C_23_ H_38_ O_5_	10.69	393.26	23-Norcholic acid	1.67	1.39	4.99	0.29	↑#	↓*	-
19	C_10_ H_8_ O	6.69	145.06	2-Naphthol	1.52	1.52	0.57	1.66	↓##	↑**	-
20	C_14_ H_14_ O_5_	10.00	263.09	3-(3-furylmethylidene)-1,5-dioxaspiro[5.5]undecane-2,4-dione	1.37	1.44	0.29	3.80	↓##	↑**	-
21	C_7_ H_7_ N O_3_	3.31	154.05	3-Hydroxyanthranilic acid	1.42	1.53	0.42	1.30	↓#	↑	o
22	C_11_ H_9_ N O_2_	6.03	186.06	3-Indoleacrylic acid	1.40	1.86	0.58	2.26	↓##	↑**	-
23	C_9_ H_13_ N O_2_	10.56	168.10	3-Methoxytyramine	1.61	1.80	0.59	1.75	↓##	↑	p
24	C_11_ H_10_ N_2_ O_2_	6.71	221.09	4-(anilinomethylidene)-3-methyl-4,5-dihydroisoxazol-5-one	1.79	1.26	0.63	1.71	↓##	↑**	-
25	C_15_ H_22_ O_3_	10.52	249.15	4-(octyloxy)benzoic acid	1.53	1.27	0.51	1.69	↓##	↑*	-
26	C_8_ H_10_ O	9.16	121.07	4-Ethylphenol	1.66	1.18	0.36	2.76	↓##	↑**	-
27	C_5_ H_11_ N_3_ O_2_	1.35	129.07	4-Guanidinobutyric acid	1.73	1.31	0.43	3.05	↓#	↑**	-
28	C_7_ H_6_ O_3_	5.73	137.02	4-Hydroxybenzoic acid	1.40	1.30	0.36	3.97	↓##	↑**	-
29	C_13_ H_10_ O_2_	10.34	197.06	4-Hydroxybenzophenone	1.54	1.46	0.52	1.69	↓##	↑**	-
30	C_7_ H_8_ O	7.75	107.05	4-Methylphenol	1.99	1.19	5.16	0.42	↑##	↓*	-
31	C_20_ H_28_ O_2_	13.68	299.20	4-Oxoretinol	1.35	1.52	0.39	2.09	↓##	↑**	-
32	C_8_ H_9_ N O_4_	5.98	182.05	4-Pyridoxic acid	1.28	1.72	0.39	3.08	↓##	↑*	-
33	C_20_ H_32_ O_4_	12.52	335.22	5(S),15(S)-DiHETE	1.46	1.30	2.06	0.36	↑#	↓*	-
34	C_11_ H_12_ N_2_ O_3_	6.55	221.09	5-Hydroxytryptophan	1.93	0.81	0.65	1.71	↓#	↑*	o
35	C_12_ H_10_ O_3_	8.86	203.07	6-Methoxy-2-naphthoic acid	1.54	1.28	8.88	0.26	↑##	↓*	-
36	C_19_ H_28_ O_3_	13.60	305.21	7α-Hydroxytestosterone	1.46	1.13	1.89	0.29	↑##	↓**	-
37	C_24_ H_48_ N O_4_	13.66	414.36	ACar 17:0	1.74	1.35	1.42	0.45	↑#	↓**	-
38	C_27_ H_52_ N O_4_	13.89	472.40	ACar 20:1	1.58	1.52	1.89	0.47	↑##	↓**	-
39	C_29_ H_56_ N O_4_	14.14	482.42	ACar 22:1	1.55	1.36	2.69	0.42	↑##	↓**	-
40	C_31_ H_60_ N O_4_	14.37	510.45	ACar 24:1	1.40	1.07	3.67	0.42	↑##	↓*	-
41	C_4_ H_6_ O_3_	1.44	101.02	Acetoacetate	0.50	1.25	1.19	1.90	↑	↑*	d, n
42	C_24_ H_40_ O_3_	14.93	375.29	Allolithocholic acid	1.48	1.49	0.73	1.41	↓##	↑*	-
43	C_7_ H_7_ N O_2_	1.55	138.06	Anthranilic acid	1.35	1.55	0.53	3.27	↓#	↑*	o
44	C_20_ H_32_ O_2_	14.16	303.23	Arachidonic acid	1.40	1.64	0.77	1.29	↓#	↑*	a
45	C_10_ H_16_ N_2_ O_3_ S	8.15	245.10	Biotin	1.45	1.01	0.58	2.31	↓##	↑*	c
46	C_20_ H_28_ O_3_	12.01	317.21	Cafestol	1.53	1.42	1.59	0.32	↑#	↓**	-
47	C_6_ H_6_ O_2_	6.94	109.03	Catechol	1.39	1.63	0.62	2.42	↓##	↑**	-
48	C_30_ H_48_ O_10_	13.59	569.33	Chenodeoxycholic acid-3-beta-D-glucuronide	1.66	1.30	4.03	0.20	↑##	↓**	-
49	C_13_ H_14_ O_5_	9.92	233.08	Citrinin	1.49	1.49	0.49	2.72	↓##	↑**	-
50	C_6_ H_12_ N_2_ O_3_	1.43	161.09	D-Ala-D-Ala	1.74	1.63	0.67	1.91	↓##	↑*	-
51	C_21_ H_21_ [_2_]H_9_ O_2_	14.59	324.29	delta8-THC-d9	1.27	1.14	2.31	0.45	↑##	↓**	-
52	C_13_ H_24_ O_2_	13.23	211.17	Delta-Tridecalactone	2.00	1.25	1.63	0.41	↑#	↓**	-
53	C_24_ H_40_ O_4_	13.05	195.14	Deoxycholic acid	1.84	1.27	6.54	0.22	↑#	↓*	-
54	C_33_ H_58_ O_14_	13.98	679.39	DGMG (18:2)	1.79	1.34	1.32	0.36	↑#	↓**	-
55	C_5_ H_11_ N O_3_	1.34	132.07	Dl-3-Hydroxynorvaline	1.81	1.05	0.57	2.17	↓##	↑**	-
56	C_5_ H_9_ N O_2_	1.54	116.07	D-Proline	1.46	1.08	0.59	1.71	↓##	↑**	-
57	C_9_ H_15_ N O_3_	9.72	186.11	Ecgonine	1.97	1.91	2.96	0.36	↑##	↓**	-
58	C_10_ H_14_ N_2_ O_2_	8.02	195.11	ethyl 3-amino-4-(methylamino)benzoate	1.45	1.09	0.73	1.69	↓##	↑**	-
59	C_15_ H_16_ N_2_ O_5_	9.50	305.11	ethyl 4-hydroxy-2-[(4-methoxyphenoxy) methyl] pyrimidine-5-carboxylate	1.34	1.28	0.21	8.76	↓##	↑**	-
60	C_38_ H_70_ O_4_	14.75	589.52	FAHFA (18:0/20:2)	1.48	1.24	1.53	0.68	↑#	↓*	-
61	C_14_ H_20_ N_2_ O_3_	9.54	265.15	Feruloyl Putrescine	1.53	1.33	0.52	4.52	↓##	↑**	-
62	C_7_ H_6_ O_4_	6.94	153.02	Gentisic acid	1.68	1.58	0.51	3.07	↓##	↑**	p
63	C_26_ H_43_ N O_6_	11.15	464.30	Glycocholic acid	1.45	1.05	57.67	0.07	↑#	↓*	-
64	C_26_ H_43_ N O_5_	11.94	448.31	Glycoursodeoxycholic acid	1.44	1.45	92.93	0.04	↑#	↓*	-
65	C_8_ H_15_ N O_3_	7.76	172.10	Hexanoylglycine	1.41	1.15	2.98	0.25	↑#	↓**	-
66	C_7_ H_16_ N_4_ O_2_	1.32	189.13	Homoarginine	1.52	1.69	0.64	1.27	↓##	↑*	-
67	C_9_ H_10_ O_4_	2.09	181.05	Homovanillic acid	1.85	2.00	0.59	1.65	↓##	↑**	p
68	C_9_ H_10_ O_2_	7.71	149.06	Hydrocinnamic acid	1.75	1.72	0.49	3.17	↓#	↑**	-
69	C_8_H_5_NO_2_	7.94	148.04	indole-5,6-quinone	1.70	0.87	1.77	0.63	↑#	↓	p
70	C_11_ H_13_ N O_4_	8.46	246.08	L-Aspartic acid β-benzyl ester	1.51	1.42	1.87	0.61	↑##	↓**	-
71	C_3_ H_7_ N O_2_ S	9.45	120.01	L-cysteine	1.25	1.50	0.86	1.24	↓	↑*	e
72	C_15_ H_11_ I_4_ N O_4_	11.90	777.70	Levothyroxine	1.56	1.26	0.59	1.86	↓#	↑	p
73	C_6_ H_9_ N_3_ O_2_	1.65	154.06	L-Histidine	1.75	1.33	0.53	1.12	↓##	↑	g
74	C_10_ H_12_ N_2_ O_3_	8.39	209.09	L-Kynurenine	1.65	0.99	0.22	4.29	↓#	↑*	o
75	C_21_ H_39_ O_7_ P	14.98	433.24	LPA 18:2	1.64	1.83	0.76	1.82	↓#	↑**	-
76	C_24_ H_50_ N O_7_ P	14.76	540.33	LPC 16:0	1.27	1.36	0.52	1.53	↓##	↑**	-
77	C_24_ H_48_ N O_7_ P	14.37	538.31	LPC 16:1	1.33	1.49	0.25	4.06	↓##	↑*	-
78	C_25_ H_50_ N O_7_ P	14.64	566.35	LPC 17:1	1.60	1.37	0.52	2.49	↓##	↑*	-
79	C_26_ H_54_ N O_7_ P	15.29	582.38	LPC 18:0	1.62	1.52	0.68	1.42	↓#	↑*	-
80	C_26_ H_48_ N O_7_ P	14.28	576.33	LPC 18:3	1.39	1.54	0.46	2.99	↓##	↑*	-
81	C_27_ H_54_ N O_7_ P	15.15	594.38	LPC 19:1	1.84	1.79	0.50	2.46	↓##	↑*	-
82	C_28_ H_50_ N O_7_ P	14.49	588.33	LPC 20:4	1.31	1.18	0.38	2.26	↓##	↑*	-
83	C_22_ H_46_ N O_7_ P	14.88	468.31	LPE 17:0	1.41	1.25	1.66	0.63	↑##	↓**	-
84	C_25_ H_50_ N O_7_ P	15.48	506.33	LPE 20:1	1.37	1.71	0.72	1.75	↓#	↑*	-
85	C_25_ H_46_ N O_7_ P	14.64	504.31	LPE 20:3	1.57	1.80	1.60	0.71	↑#	↓*	-
86	C_22_ H_43_ O_9_ P	13.68	481.26	LPG 16:1	1.51	1.21	0.44	1.97	↓#	↑*	-
87	C_24_ H_44_ N O_9_ P	13.83	520.27	LPS 18:2	1.79	1.75	2.14	0.47	↑##	↓**	-
88	C_4_ H_9_ N O_3_	1.27	120.07	L-Threonine	1.65	1.01	0.41	2.89	↓##	↑**	-
89	C_11_ H_12_ N_2_ O_2_	6.70	205.10	L-Tryptophan	1.55	1.69	0.62	1.56	↓#	↑	o
90	C_9_ H_11_ N O_3_	1.98	180.07	L-Tyrosine	1.59	1.27	0.57	1.44	↓##	↑	k, l, p
91	C_24_ H_48_ N O_7_ P	14.66	494.33	Lysopc 16:1	1.63	1.18	0.50	1.92	↓#	↑*	-
92	C_25_ H_52_ N O_7_ P	15.29	508.34	Lysopc 17:0	1.60	1.59	0.68	1.42	↓#	↑*	-
93	C_26_ H_54_ N O_7_ P	15.29	522.36	LysoPC 18:0	1.50	1.22	0.70	1.36	↓#	↑*	-
94	C_28_ H_50_ N O_7_ P	14.49	542.32	Lysopc 20:4	1.34	1.22	0.43	2.22	↓##	↑*	-
95	C_5_ H_11_ N O_2_ S	1.43	150.06	Methionine	1.72	1.51	0.60	1.60	↓##	↑**	e
96	C_12_ H_15_ N O_4_ S	9.44	268.06	N-(1,1-Dioxotetrahydro-1H-1λ6-thiophen-3-yl)-4-methoxybenzamide	1.43	1.17	0.18	13.69	↓##	↑**	-
97	C_9_ H_20_ N_2_ O_2_	1.29	189.16	N6,N6,N6-Trimethyl-L-lysine	1.13	1.26	0.81	1.43	↓	↑*	h
98	C_6_ H_6_ N_2_ O	1.83	123.06	Nicotinamide	1.01	1.48	0.73	0.65	↓	↓*	i
99	C_19_ H_38_ O_2_	14.91	297.28	Nonadecanoic acid	1.73	1.46	1.66	0.58	↑#	↓**	-
100	C_13_ H_16_ N_2_ O_4_	7.75	265.12	N-Phenylacetylglutamine	1.41	1.32	5.81	0.26	↑#	↓*	-
101	C_20_ H_39_ N O_2_	14.89	326.30	Oleoyl ethanolamide	1.43	1.09	1.69	0.54	↑##	↓**	-
102	C_6_ H_8_ O_5_	1.20	159.03	Oxoadipic Acid	0.07	1.64	0.98	1.97	↓	↑**	h
103	C_46_ H_86_ N O_7_ P	15.79	796.62	PC (14:0e/24:4)	1.80	1.46	1.68	0.51	↑#	↓*	-
104	C_49_ H_86_ N O_8_ P	13.84	848.63	PC (21:2/20:4)	1.36	1.45	1.30	0.73	↑##	↓**	-
105	C_26_ H_50_ N O_8_ P	13.55	536.33	PC (5:0/13:1)	1.75	1.78	0.33	4.04	↓##	↑**	-
106	C_23_ H_46_ N O_8_ P	13.20	474.33	PC (7:0/8:0)	1.28	1.02	2.07	0.38	↑##	↓**	-
107	C_26_ H_52_ N O_8_ P	13.83	538.35	PC (9:0/9:0)	1.62	1.92	0.36	3.17	↓##	↑**	-
108	C_40_ H_80_ N O_8_ P	16.96	734.57	PC(16:0/16:0)	1.00	1.56	0.71	1.59	↓	↑**	a, f
109	C_10_ H_11_ N O_3_	8.13	194.08	Phenylacetylglycine	1.59	1.70	4.26	0.29	↑##	↓*	k
110	C_9_ H_6_ O_2_	8.80	147.04	Phenylpropiolic acid	1.68	1.81	0.45	4.29	↓##	↑**	-
111	C_9_ H_8_ O_3_	1.98	163.04	Phenylpyruvic acid	1.41	0.10	0.66	1.42	↓#	↑	k, l
112	C_18_ H_20_ N_2_ O_3_	12.96	313.15	Phe-Phe	1.66	1.67	2.13	0.38	↑#	↓*	-
113	C_41_ H_79_ O_13_ P	15.91	809.52	Phosphatidylinositol-1,2-dipalmitoyl	1.92	1.70	0.36	2.36	↓#	↑*	-
114	C_5_ H_14_ N O_4_ P	14.43	184.07	Phosphocholine	1.67	1.57	0.77	1.34	↓##	↑**	-
115	C_5_ H_14_ N O_4_ P	14.43	184.07	Phosphocholine	1.67	1.57	0.77	1.34	↓##	↑**	f
116	C_11_ H_16_ N_2_ O_2_	9.04	209.13	Pilocarpine	1.60	1.61	0.32	3.44	↓##	↑**	-
117	C_10_ H_19_ N O_4_	10.36	218.14	Propionyl-L-carnitine	1.96	1.12	2.39	0.52	↑##	↓*	-
118	C_20_ H_30_ O_4_	11.77	317.21	Prostaglandin A2	1.98	1.91	2.18	0.33	↑##	↓**	-
119	C_20_ H_32_ O_5_	11.09	351.22	Prostaglandin D2	1.17	1.26	1.81	0.42	↑	↓*	a
120	C_20_ H_36_ O_5_	11.24	355.25	Prostaglandin F1α	1.38	1.55	2.48	0.25	↑##	↓**	-
121	C_20_ H_32_ O_6_	13.13	369.23	Prostaglandin G2	0.57	1.36	1.50	0.14	↑	↓*	a
122	C_17_ H_33_ N_7_ O_4_	11.18	400.27	RPK	1.46	1.42	1.61	0.54	↑#	↓**	-
123	C_17_ H_28_ O_6_	13.09	329.20	Spiculisporic Acid	1.72	1.55	1.49	0.67	↑#	↓*	-
124	C_26_ H_45_ N O_8_ S_2_	11.75	562.25	Taurolithocholic acid 3-sulfate	1.61	1.43	8.19	0.19	↑##	↓**	-
125	C_20_ H_34_ O_6_	11.00	369.23	Thromboxane B2	1.38	1.05	1.68	0.43	↑#	↓*	-
126	C_15_ H_22_ N_2_ O_18_ P_2_	1.34	579.03	UDP-D-glucuronate	1.57	0.76	0.60	1.67	↓#	↑	b, j
127	C_15_ H_24_ N_2_ O_17_ P_2_	1.33	565.05	Uridine 5'-diphospho-D-glucose	1.70	1.54	1.37	0.76	↑#	↓*	-
128	C_17_ H_27_ N_3_ O_17_ P_2_	1.34	606.08	Uridine 5'-Diphospho-N-Acetylgalactosamine	1.79	1.78	0.45	2.25	↓##	↑**	-
129	C_17_ H_20_ N_4_ O_6_	7.92	377.15	Vitamin B2	1.33	1.70	0.37	3.02	↓##	↑**	m
130	C_5_ H_12_ O_5_	1.33	151.06	Xylitol	1.37	0.13	1.88	0.96	↑#	↓	j

**Note:** Control group (C), Model group (M), Crocin40 group (CR), n=6 per group.Name of pathways a: Arachidonic acid metabolism, b: Ascorbate and aldarate metabolism, c: Biotin metabolism, d: Butanoate metabolism, e: Cysteine and methionine metabolism, f: Glycerophospholipid metabolism, g: Histidine metabolism, h: Lysine degradation, i: Nicotinate and nicotinamide metabolism, j: Pentose and glucuronate interconversions, k: Phenylalanine metabolism, l: Phenylalanine, tyrosine and tryptophan biosynthesis, m: Riboflavin metabolism, n: Synthesis and degradation of ketone bodies, o: Tryptophan metabolism, p: Tyrosine metabolism.

#: *p<*0.05, compared with control group; ##: *p<*0.01, compared with control group; *: *p<*0.05, compared with model group; **: *p<*0.01, compared with model group.

## Data Availability

The data and supportive information are available within the article.

## LIST OF ABBREVIATIONS

DN: Diabetic Nephropathy
FBG: Fasting Blood Glucose
GSH-Px: Glutathione Peroxidase
HE: Hematoxylin and Eosin
HFD: High-Fat Diet
MDA: Malondialdehyde
PAS: Periodic Acid-Schiff
PCA: Principal Component Analysis
QC: Quality Control
ROS: Reactive Oxygen Species
STZ: Streptozotocin
SOD: Superoxide Dismutase
